# Development of A Japanese Version of the Family Poly-Victimization Screen (FPS-J)

**DOI:** 10.3390/ijerph20043142

**Published:** 2023-02-10

**Authors:** Sachiko Kita, Kaori Baba, Riho Iwasaki-Motegi, Emiko Kishi, Kiyoko Kamibeppu, Wenche Karin Malmedal, Ko Ling Chan

**Affiliations:** 1 Department of Family Nursing, Division of Health Sciences & Nursing, Graduate School of Medicine, The University of Tokyo, Tokyo 113-0033, Japan; 2 Global Nursing Research Center, Graduate School of Medicine, The University of Tokyo, Tokyo 113-0033, Japan; 3 Department of Health Policy, National Center for Child Health and Development, Tokyo 157-0074, Japan; 4 Research Centre for Social Science and Medicine, Tokyo Metropolitan Institute of Medical Science, Tokyo 156-8506, Japan; 5 Section of Public Health Nursing Research Department of Health Promotion, National Institute of Public Health, Saitama 351-0197, Japan; 6 Department of Community Nursing, Toho University, Tokyo 143-8540, Japan; 7 Graduate Programs in Family Nursing, International University of Health and Welfare, Tokyo 107-8402, Japan; 8 Department of Public Health and Nursing, Faculty of Medicine and Health Sciences, Norwegian University of Science and Technology, N-7491 Trondheim, Norway; 9 Department of Applied Social Sciences, The Hong Kong Polytechnic University, Hong Kong, China

**Keywords:** child abuse, development, elder abuse, family poly-victimization, intimate partner violence, scale, validity

## Abstract

This study developed a Japanese version of the Family Poly-Victimization Screen (FPS-J) and assessed its validity. A cross-sectional study using self-report questionnaires was conducted with parents of children in Tokyo, Japan, from January to February 2022. To test the validity of the FPS-J, we used the Japanese versions of the revised Conflict Tactics Scale Short Form (J-CTS2SF) as the gold standard for intimate partner violence (IPV), the Conflict Tactics Scale Parent-Child (J-CTS-PC) for child abuse (CAN), the Conflict Tactics Scale (J-MCTS) for elder abuse, the K6-J for depression and anxiety, the PCL5-J for post-traumatic stress disorder, and the J-KIDSCREEN for Health-related Quality of Life among children. Data from 483 participants (response rate: 22.6%) were used. The J-CTS2SF and J-CTS-PC scores were significantly higher among the IPV/CAN-victim groups than in the non-victimized groups classified by the FPS-J (*p* < 0.001). The JMCTS scores did not differ significantly between the victim and non-victim groups (*p* = 0.44), but the PCL5-J, K6-J, and J-KIDSCREEN-10 scores were either significantly higher or lower among victims of violence than among the non-victim groups (*p* < 0.05). This study suggests the validity of parts of the FPS-J, especially the IPV against respondents and CAN by respondents.

## 1. Introduction

Family violence, such as intimate partner violence (IPV), child abuse and neglect (CAN), and elder abuse (EA), is a common and serious health and social issue worldwide. Approximately one in every three to four adults, one in every three children, and one in every six older persons worldwide have experienced violence and abuse [1,2,3]. In Japan, 31.3% of women and 19.9% of men have experienced IPV during their lifetime, and the number of consultations for IPV at Spousal Violence Counseling and Support Centers/Female Counseling Offices (SVCSCs/FCOs) reached a maximum by 2020 [4]. Although the national prevalence of CAN has not been reported, a previous study reported that 20.0% of infants in Japan have experienced CAN [5]; the number of consultations regarding CAN at Children and Family Support Centers/Child Counseling Centers (CFSCs/CCCs) also reached a maximum in 2020 [6]. Although the prevalence of EA has not been reported by the government or previous studies in Japan, the number of consultations on EA by family caregivers for formal facilities, such as Community Comprehensive Support Centers (CCSs), reached its highest number in the last decade in 2020 [6]. Such family violence has several lifelong adverse health outcomes, such as physical injuries, post-traumatic stress disorder (PTSD), depression, anxiety, unintended pregnancy, alcohol and drug misuse, cognitive decline, obesity, chronic heart and digestive problems, and enormous economic costs throughout the victims’ lifetimes [1,4,5,7,8]. Thus, the development and implementation of effective interventions and policies to prevent and terminate family violence are urgently needed to prevent adverse health outcomes and socioeconomic damage.

The co-occurrences and multiple experiences of IPV, CAN, and/or EA in households have been often observed in clinical settings and reported by recent studies [9,10,11,12]. Such a phenomenon, defined as “family poly-victimization” [9], has received increasing attention due to its more severe impacts on health and the intergenerational transmission of violence among victims than those from a single type of violence. Recent studies have reported that two or more types of violence victimization in households, such as the co-occurrence of IPV and CAN and the co-occurrence of IPV, CAN, and EA, could lead to more severe psychological and physical trauma and chronic adverse health outcomes, such as PTSD, depression, serious injury, surgery, heart murmurs, asthma, addictive disorders (e.g., alcohol abuse), daily medication requirements, and allergies [9,11,13,14]. A Japanese study also identified pregnant women with multiple experiences of IPV and childhood abuse as having a higher risk of CAN after childbirth than those with a single experience of IPV or childhood abuse [12]. These findings suggest that these three types of violence in a family often strongly influence each other both simultaneously and inter-generationally. Thus, assessment and interventions for only a single type of violence are insufficient; it is important to understand the overall picture of the effects on the entire family by assessing violence from the perspectives of all family members and developing new policies, systems, and interventions for preventing and terminating existing and/or future cycles of such violence.

Although the number of studies focusing on family poly-victimization has increased recently, most have focused on poly-victimization involving two types of violence: IPV and CAN [12,15] or IPV and EA [15,16]. One of the main reasons for the difficulty of research focusing on family poly-victimization involving all three types (i.e., IPV, CAN, and EA) is the absence of a method to assess these three types simultaneously, more easily and efficiently. Although several assessment tools to assess IPV, CAN, and EA individually have been created and applied in previous studies, such as the revised Conflict Tactics Scales Short Form for IPV against respondent (CTS2SF) [17,18], Conflict Tactics Scale Parent-Child for CAN by a parent [17,19], and Modified Conflict Tactics Scale (MCTS) for EC by a family caregiver [20,21], each scale has numerous items (approximately 10 to 30 items), including items asking directly about the detailed experiences of violence, that may impose physical and psychological burdens on respondents, especially if these scales are used simultaneously to assess poly-victimization involving all three types of violence. In Japan, the majority of caregivers of elderly people who need care are family members (70.8%), and 31.5% are a child and child’s partner of the elderly who are living together [22]; thus, identifying family victimization, including EA, from the perspective of the elderly’s children, may be more suitable and feasible in the Japanese context. Thus, the development of an assessment tool to evaluate this three-fold family poly-victimization more easily and effectively is needed to clarify the overall picture of family poly-victimization with less burden on respondents. In addition, it is more feasible to ask children’s parents and the elderly to assess family poly-victimization, although this method may lead to limitations and information bias. It is difficult to collect information directly from children and the elderly, possibly because of their cognitive abilities and excessive burdens.

The Family Poly-victimization Screen (FPS) was recently developed in Hong Kong by Chan et al. [10]. The FPS has a limited number of items (11) to assess IPV, CAN, and EA simultaneously and/or inter-generationally, and can be used for parents with children aged less than 19 years. The FPS has four modules: (a) violence against respondents (IPV victimization), (b) violence against partners (IPV perpetration), (c) violence against children (CAN), and (d) violence against parents (EA) [10]. The IPV victimization and perpetration modules consist of three items covering psychological, physical, and sexual violence; four items assessing psychological, physical, and sexual abuse and neglect for the CAN module; and five items assessing psychological, physical, sexual, and financial abuse and neglect for the EA module. In addition, each item includes examples of violent acts, such as yelling at, humiliating, or cursing the respondent and isolating the respondent from friends/family for psychological violence against the respondent; beating, shaking, or choking a child for physical child abuse; sexual acts or attempts to do so with the child (whether the child is willing) for sexual child abuse; and taking money without the elder’s permission and cheating the elder of property for financial EA. Respondents respond with a “yes” or “no”; if they answer “yes”, they are asked to answer additional questions regarding who committed such violence and when it happened. Because such a scale useful for measuring family poly-victimization more efficiently and comprehensively and adapted to Japanese culture has not been developed, actual situations, such as the prevalence of family poly-victimization and its related factors, have not been identified accurately, and an effective policy, system, and interventions to prevent and terminate family poly-victimization have not yet been developed.

This study aimed to develop a Japanese version of the FPS (J-FPS) and to assess its validity using external criteria.

## 2. Materials and Methods

### 2.1. Study Design

A cross-sectional study using self-report online questionnaires was conducted involving parents of children aged less than 19 years.

### 2.2. Study Setting and Period

This study was conducted in a city located in the suburbs of Tokyo, Japan, between January and February 2022.

### 2.3. Participants

Parents living in the city during the study period were considered potential participants. Eligible participants were selected according to the following criteria: (1) age from 20 to 65 years and (2) living together with a child aged 0–19 years. Parents who seemed to have an insufficient understanding of Japanese were excluded after confirming whether the eligible participants had given their Japanese names.

### 2.4. Procedure

Eligible participants were randomly selected from the Basic Resident Register (BRR) in the city and mailed a flyer with brief explanations (e.g., the study aims and design, the recruitment criteria and method) and the QR code and URL to access the study questionnaires. When they accessed the QR code or URL, they received detailed explanations of this study, including ethical considerations regarding the reporting policies for detecting IPV, CAN, and EA throughout their responses; they were then asked to consent to participate in this study. After consenting, they were asked to complete the questionnaires online, which took approximately 30 min, in a private room/area by themselves. Participants were provided with a 500-yen gift (USD 5) card, using their email address as an incentive to participate in the study. The major reason for this recruitment method was to assess the situation of family violence among the general population to increase the generalizability and usability of the results.

### 2.5. Translation and Development Process of the FPS-J and J-MCTS

Translation from English to Japanese was implemented according to the guidelines for translating measurement tools [23]. First, the developer of the original version of the FPS (K. L. Chan, a co-author of this study) was contacted and asked to approve the translation and development of the FPS-J. Once approval was received, two researchers (SK and KB) independently translated the FPS into Japanese. Both translators were proficient in English and in the field of family violence. After completing the forward translation, our team of five researchers focused on family violence and maternal health (one for IPV, SK; two for CAN, KB, and KK; one for maternal health, RIM; and two for EA, EK, and WM) carefully discussed the translations and developed the proposal for the FPS-J. Cognitive interviews were conducted with nine professionals who provided support to victims of IPV, CAN, and EA in formal and informal facilities (three of whom had experienced violence, such as IPV and/or CAN), such as CFSC, SVCSC, FCO, CCSC, or NPO IPV and CAN shelters, and with two survivors of IPV and/or CAN to confirm the content and face validity of the FPS-J. Based on the data from the cognitive interviews, the wording, content, and cultural adaptations of the FPS-J were carefully discussed by the research team. In addition, the definitions of IPV, CAN, and EA and their examples were carefully organized and discussed from the perspectives of victims according to the results of the cognitive interviews, Japanese laws, and the experiences and knowledge of the researchers. The following parts of the FPS-J were changed from the original version to reflect the actual experiences and situations among survivors of family violence in Japan, adapting more to the Japanese culture: (1) avoiding the use of advanced terms and adding *furigana* (indications of pronunciation) to *kanji* (Chinese characters) because victims/perpetrators of violence may include those with lower education and cognitive ability; (2) adding examples of violence/abuse to indicate more common kinds of violence in Japan, such as inappropriate sexual contact in bathrooms and forcing the child to bathe together for early adolescent children for sexual CAN, witnessing IPV and acting to prevent education for psychological CAN, dousing with cold water for physical abuse, and unnecessary restriction of physical freedom for physical EA; (3) adding financial violence to the items of violence toward respondents and partners, as Japanese victims of IPV tend to experience more financial violence, such as not being given living money and being prohibited from working; and (4) adding response items to ask frequencies of each violence within the past six month: once (1) to over 10 times (5), to increase the usability of their responses for analysis and interpretation and to identify the degree of urgency of each form of violence. The reconciled translations with the above changes from the original version were back-translated from Japanese to English by a professional translator, and the developer of the original version sent the back-translated FPS-J for review. The development of the FPS-J was deemed complete after approval.

In addition, because no validated scales for EA exist, we developed the Japanese version of the MCTS (J-MCTS) through the same process of translation and development as the FPS-J: (1) receiving approval by the developer of the original version of the MCTS (Scott Richard Beach); (2) independent translations from English to Japanese by two researchers; (3) discussions by the research team to create the proposal of the FPS-J; (4) cognitive interviews with nine professionals and survivors of family violence; (5) discussion by the research team and development of the J-MCTS; (6) back-translations from English to Japanese by a professional translator; and (7) asking the developer of the original version to review and approve the J-MCTS.

### 2.6. Measures

**Demographics.** Data on age, sex, nationality, marital/educational/working status, annual income, history of mental/physical illness (and, if yes, whether the mental/physical illness has continued to now), number of family members living together/children, and age of children were collected.

**FPS-J.** The FPS-J has four modules: (a) violence against respondents (IPV against respondents), (b) violence against partners (IPV against partners), (c) violence against children (CAN), and (d) violence against parents (EA) (see Appendix A). The violence against respondents and partners module consists of four items: psychological, physical, sexual, and financial violence. The CAN module comprises four items assessing psychological, physical, and sexual abuse and neglect, whereas the EA module includes five items assessing psychological, physical, sexual, and financial abuse and neglect. All 17 items were asked using dichotomous questions, such as “Have you ever been psychologically hurt? (violence against the respondent)”, “Have your partner been physically hurt? (violence against your partner)”, “Has your child been sexually assaulted (CAN)?”, and “Has your parent been neglected (EA)?” If respondents answered “yes”, they were asked who committed the violence (perpetrators) and when it happened (timeframes). Regarding the time frames, if they answered “within six months”, they were asked the frequency: once, two to three times, four to nine times, and more than ten times to assess the urgency of violence. Before they answered the CAN module, they were asked to choose a child whose birthday was closest to that day’s date (the date this survey was answered), in the case of multiple children, to encourage them to select a child randomly to reduce the burden on respondents, similar to the original version of the FPS. Before the EA module, they were asked to answer questions about their surviving parents, including parents-in-law; if they did not have any surviving parents, this module was automatically skipped from the web system. In particular, we tested the validities of the items of the FPS-J regarding IPV against respondents (psychological, physical, and sexual violence), CAN (psychological, physical, and sexual abuse and neglect), and EA (psychological and physical abuse) in this study because of the lack of scales assessing the other forms of violence.

**IPV**. The items of the Japanese version of the revised Conflict Tactics Scale Short Form (J-CTS2SF) [17,18,24] were used as external criteria to measure IPV. The J-CTS2SF has 10 items assessing the degree of victimization by IPV during the past 12 months and a five-factor structure: negotiation (2 items), psychological aggression (2 items), physical assault (2 items), injuries (2 items), and sexual coercion (2 items). Respondents scored these items on an eight-point scale: 1 = *once*, 2 = *twice*, 3 = *3–5 times*, 4 = *6–10 times*, 5 = *11–20 times*, 6 = *more than 20 times*, 7 = *not in the past year, but it did happen before*, and 8 = *never happened*. The validity and reliability of the J-CTS2SF have been confirmed [18]. The cut-off points of the J-CTS2SF have not yet been identified. Eight items of the four factors of psychological aggression, physical assault, injuries, and sexual coercion were used, and the scores were re-coded and re-ordered according to rank (0 = *never happened* and *not in the past year, but it did happen before*, 1 = *not in the past year, but it did happen before*, 2 = *once*, 3 = *twice*, 8 = *3–5 times*, 15 = *6–10 times*, 25 = *11–20 times*, and 7 = *more than 20 times*) according to the instructions of the J-CTS2SF for the analyses in this study [17,18]. Higher scores indicated a higher severity of IPV in the past year. Cronbach’s alphas of the subscales of the J-CTS2SF in this study were: α = 0.26 (Psychological aggression), α = 0.86 (Physical assault), α = 0.03 (Injuries), and α = 0.66 (Sexual coercion).

**CAN.** The Japanese version of the Conflict Tactics Scale-Parent Child (J-CTS-PC) [17,19] was used as external criteria to measure CAN. The J-CTS-PC has 29 items for seven factors: nonviolent discipline with four items, psychological aggression with five items, minor physical assault (corporal punishment) with six items, severe physical assault with three items, extreme physical assault with four items, neglect with five items, and sexual abuse with two items. These questions assessed the degree of CAN during the past 12 months as reported by the respondents using an eight-point scale (1 = *once*, 2 = *twice*, 3 = *3–5 times*, 4 = *6–10 times*, 5 = *11–20 times*, 6 = *more than 20 times*, 7 = *not in the past year*) [17]. This study used six factors with 25 items: Psychological aggression, physical assault_minor, physical assault_severe, physical assault_extreme, neglect, and sexual abuse. Before answering the items, the respondents were instructed to identify the same child chosen for the CAN module of the FPS-J. The J-CTS-PC scores were re-coded and re-ordered by rank, similar to the J-CTS2SF. Higher scores indicated a higher severity of CAN. The validity and reliability of the CTS-PC have been previously reported [17,19]. The cut-off points of the J-CTS-PC were not examined. Cronbach’s alphas of the subscales J-CTS-PC in this study were as follows: α = 0.63 (Psychological aggression), α = 0.80 (Physical assault_total), α = 0.81 (Physical assault_minor), α = 0.26 (Physical assault_severe), α = 0.59 (Physical assault_extreme), α = 0.26 (Neglect), and α = 0.59 (Sexual abuse).

**EA.** The Japanese version of the Modified Conflict Tactics Scale (J-MCTS) [20,21] was used as an external criterion to measure EA. The J-MCTS has 10 items for respondents on EA during the past three months and a two-factor structure: psychological mistreatment (five items) and physical mistreatment (five items) using a five-point Likert scale from 0 (*None*) to 4 (*Always*). Higher scores indicate a higher severity of EA. Respondents were asked to answer with the same parent as in the EA module of the FPS-J. The validity and reliability of the MCTS have been confirmed [21]. The cut-off points for the MCTS have not yet been identified. Cronbach’s alphas of the subscales J-MCTS in this study were α = 0.77 (Psychological mistreatment) and α = 0.99 (Physical mistreatment).

**Depression and anxiety**. The Japanese version of the Kessler 6 (K6-J) was used to assess psychological distress, particularly depression and anxiety, during the past 30 days [25,26]. The K6-J has six items and a 5-Likert scale from 0 (*None*) to 4 (*Always*) and a one-factor structure. A higher K6-7-J score indicates a greater degree of psychological distress (i.e., depression and anxiety). The cut-off points of this scale were as follows: >5, psychological distress; >8, mood and anxiety disorders; and >12, severe mental illness. The reliability and validity of the K6-J have been confirmed [25,26]. The reliability of the K6-J was 0.89 in this study.

**Post-traumatic stress disorder**. The Japanese version of the PCL5 (PCL5-J) was used to evaluate PTSD during the past month using 20 items [27,28,29]. The PCL5-J has four factors: intrusion with five items, avoidance with two items, negative alterations in cognition and mood with seven items, and arousal and reactivity with five items, all scored on a four-point Likert scale from 0 (*Not at all*) to 4 (*Extremely*), where higher scores indicate more severe symptoms of PTSD. The PCL5-J has a cut-off point of 31 for severe PTSD symptoms [27,28,29]. The reliability and validity of the PCL5-J have been confirmed [27]. The reliability of the K6-J was 0.94 in this study.

**QOL among children**. The Japanese version of the KIDSCREEN-10 (J-KIDSCREEN-10) was used to assess the Health-related Quality of Life (HQOL) of their child aged from 8 to 18 years of age [30,31]. The J-KIDSCREEN-10 has a single-factor structure and contains 10 items scored on a six-point Likert scale from 0 (*Not at all*) to 5 (*Always*), where a higher score indicates better HQOL. The participants were asked to identify the child chosen for the CAN module of the FPS. The validity and reliability of the J-KIDSCREEN-10 have been confirmed [30]. Because of the limited age available for using the J-KIDSCREEN-10 (i.e., 8–18 years old), respondents were asked if their child was older than 8 years old; if yes, they were asked to answer the J-KIDSCREEN-10, and if not, the J-KIDSCREEN-10 was automatically skipped. The Cronbach’s alpha for the Japanese version of the KIDSCREEN-10 was 0.81.

### 2.7. Statistical Analyses

First, descriptive statistics for demographics and FPS-J, J-CTS2SF, J-CTS-PC, J-MCTS, K6-J, PCL5-J, and J-KIDSCREEN-10 scores were calculated as *n* (%) or mean (*SD*). Regarding the criterion-related validity of the FPS-J, the scores of the subscales of the J-CTS2SF, J-CTS-PC, and J-MCTS were compared between the victimized and non-victimized groups as classified by the dichotomous questions of the three modules of the FPS-J: IPV against respondents (psychological, physical, and sexual violence), CAN by respondents (psychological, physical, and sexual abuse and neglect), and EA (psychological and physical abuse) using Welch’s *t*-test. In addition, the scores of the K6-J, PCL5-J, and J-KIDSCREEN-10 were compared between the two groups, victimized and non-victimized, as classified by the two modules: violence against the respondent (psychological, physical, sexual, and financial violence) and CAN by any family member (psychological, physical, and sexual abuse and neglect) using Welch’s *t*-test to examine the external validity of the FPS-J. Analyses were conducted using the Statistical Package for Social Science (SPSS) version 20.0.

### 2.8. Ethical Considerations

This study protocol for the cognitive interviews and cross-sectional study using self-administered online questionnaires was approved by the Ethical Committee of the Graduate School of Medicine, University of Tokyo (2020197NI), and the Ethical Committee of the National Center for Child Health and Development (2021-109), respectively.

As this study collected information on IPV, CAN, and EA, researchers followed a reporting policy for each type of violence according to the Act on Prevention of Spousal Violence and Protection of Victims [32], Prevention of CAN [33], Prevention of EA, Support for Caregivers of Elder Persons, and Other Related Matters [34] of Japan. These acts specify the mandatory reporting of a risk of CAN to a CFSC/Child Counseling Center located in a region and of severe IPV and EA that might threaten the victim’s life to an SVCSC, FCO, or CCSC. Thus, all participant responses were carefully reviewed, and cases indicating IPV, CAN, and EA were noted weekly by two researchers (SK and KB). Regarding cases indicating CAN, the researcher (SK) immediately reported and shared detailed responses regarding CAN and the respondents’ names and addresses to a CFSC in the city where they agreed to cooperate with this study. Regarding cases indicating IPV and EA, those with a high priority for reporting were selected through discussions at our monthly research team meetings. After the meetings, the researcher (SK) reported and shared detailed responses regarding EA and the respondents’ names and addresses with the CFSC and CCSC and asked them to support the cases reported. Cases of IPV were reported to the CFSC and not to the SVCSC/FCO because the CFSC has a professional IPV counselor and supports victims and their children exposed to IPV. This is because the Act on Prevention of CAN defines IPV in a household with a child as CAN. The reporting protocols in this study were discussed and decided upon with the CFSC and CCSC in the city, and were approved by the IRB. Furthermore, participants were provided with detailed explanations of these reporting policies before they were asked to consent to participate in this study. Information regarding the social resources available for IPV, CAN, and EA was provided on the last page of the questionnaire. To ensure the safety and privacy of participants, they were asked to complete the questionnaires online.

## 3. Results

A total of 2133 people were recruited and sent the flyer for this study, of whom 483 agreed to participate and completed the questionnaires online. The response rate was thus 22.6%.

### 3.1. Demographic

The mean age of the participants was 41.71, and the majority were married (93.2%), working (79.5%), and had graduated from higher education than a technical or junior college (81.5%). All of them were Japanese (100%), about half of them were female (52.2%), and the majority had an annual income over USD 50,000 (82.6%). Regarding their history of mental and physical illness, 52 participants (10.8%) had experienced a mental illness; of these, 37 participants (26.9%) reported the mental illness as current; moreover, 80 participants (16.6%) had experienced physical illness, of whom 50 (62.5%) reported a physical illness as current (Table 1).

In addition, the mean numbers of family members living together and of children were 3.95 and 1.90, respectively. The mean ages and rates of males by birth order were as follows: 1st (*n* = 483), 11.18 and 48.0%; 2nd (*n* = 327), 9.70 and 55.7%; 3rd (*n* = 96), 8.05 and 53.1%; 4th (*n* = 11), 8.00 and 27.3 (Table 1).

### 3.2. Descriptions of the FPS-J

Regarding violence against respondents measured by the FPS-J, the rates of the victimization in their lifetime and the perpetration by their partner (IPV) over the past year of psychological, physical, sexual, and financial violence were psychological, 48.4% and 23.6%; physical, 24.2% and 2.3%; sexual, 8.5% and 1.9%; and economic, 9.5% and 1.7%, respectively (Table 2).

Regarding violence against a child selected randomly, the mean age was 9.70, and the rates of the victimization in their lifetime and the perpetrations by the participants for the past year of psychological, physical, and sexual abuse and neglect were as follows: psychological, 35.0% and 6.4%; physical, 14.5% and 5.6%; sexual, 1.0% and 0.0%; and neglect, 0.4% and 0.0% (Table 2).

Regarding violence against parents, 468 participants (96.9%) answered that a parent was still living, and a majority of them answered regarding their mothers (72.9%). The majority of the parents were over 65 years old (74.4%), and one-third of them were over 75 years old (29.5%). The rates of victimization in their lifetime and perpetration by the participants in the past year of psychological, physical, sexual, and financial abuse and neglect were as follows: psychological, 23.7% and 2.3%; physical, 8.1% and 0.6%; sexual, 0.2% and 0.0%; economic, 6.0% and 0.0%; and neglect, 0.9% and 0.0%, respectively (Table 2).

### 3.3. Descriptions of the Variables Used as External Criteria

The means (SDs) of the subscales of the J-CTS2SF for IPV, J-CTS-PC for CAN, and J-MCTS for EA were J-CTS2SF: psychological aggression = 2.19, physical assault = 0.27, injuries = 0.08, and sexual coercion = 0.32; J-CTS-PC: psychological aggression = 10.16, physical assault/injuries = 3.98, physical assault_minor = 2.98, physical assault_severe = 0.90, physical assault_extreme = 0.46, sexual coercion = 3.74, and neglect = 0.02; J-MCTS: psychological mistreatment = 0.29, physical mistreatment = 0.06 (Table 3).

In addition, the mean (SDs)/rates of the total/subscales of the PCL5-J (PTSD), K6-J (depression/anxiety), and J-KIDSCREEN-10 (child’s HQOL) were as follows: PCL5-J: mean = 6.69 and, rate of PTSD = 4.8%; K6-J: mean = 2.52 and the rates of psychological distress, mood/anxiety disorder, and severe mental disorder were 18.8%, 7.0%, and 2.7%, respectively; J-KIDSCREEN-10: mean = 41.12.

### 3.4. Validity of the FPS-J

Compared with the scores of the J-CTS2SF, J-CTS-PC, and J-MCTS as the gold standards between the two groups classified by the FPS-J regarding victimized and non-victimized IPV/CAN/EA respondents for the past year, all the scores of the subscales of the J-CTS2SF were significantly higher among the victimized group than the non-victimized group (Psychological aggression: 7.46 vs. 0.56, *p* < 0.001; Physical assault: 4.00 vs. 1.77, *p* < 0.001; Injuries: 1.27 vs. 0.05, *p* < 0.001; Sexual coercion: 8.89 vs. 0.12, *p* < 0.001). In addition, all the scores of the subscales of the J-CTS-PC were significantly higher among the victimized group than the non-victimized group (Psychological aggression: 21.32 vs. 6.63, *p* < 0.001; Physical assault/injuries: 20.21 vs. 3.11, *p* < 0.001; Physical assault_minor: 14.44 vs. 2.36, *p* < 0.001; Physical assault_severe: 5.04 vs. 0.66, *p* < 0.001; Physical assault_extreme: 2.54 vs. 0.34, *p* < 0.001), except that two subscales (i.e., sexual coercion and neglect) could not be compared because no cases were classified into the victimized groups by the FPS-J. Regarding EA, the scores of one subscale of the J-MCTS (psychological mistreatment) were not significantly different between the two groups (Psychological mistreatment: 0.55 vs. 0.26, *p* = 0.44), and the scores of the other subscale (physical mistreatment) could not be compared because no cases were classified into the victimized group by the FPS-J (Table 4).

The results of comparing the scores of the PCL5-J, K6-J, and J-KIDSCREEN-19 between the two groups classified as victimized and non-victimized in their lifetime by the FPS-J (especially violence against respondent and violence against a child) showed that the scores of the PCL5-J and K6-J were significantly higher among the victimized groups of psychological/physical/sexual/financial violence against respondent than the non-victimized groups: PCL5-J: Psychological violence: 9.04 vs. 4.39, *p* < 0.001; Physical violence: 9.99 vs. 5.43, *p* < 0.001; Sexual violence: 13.31 vs. 6.02, *p* = 0.007; Financial violence: 13.54 vs. 5.90, *p* = 0.001; K6-J: Psychological violence: 3.29 vs. 1.74, *p* < 0.001; Physical violence: 3.37 vs. 2.19, *p* = 0.006; Sexual violence: 4.54 vs. 2.32, *p* = 0.01; Financial violence: 4.80 vs. 2.28, *p* = 0.003, respectively. In addition, the scores of the J-KIDSCREEN-10 were significantly lower among the groups victimized by psychological, physical, and sexual abuse of children than among the non-victimized groups: Psychological abuse: 39.56 vs. 42.74, *p* < 0.001; Physical abuse: 39.30 vs. 41.79, *p* = 0.002; sexual abuse: 25.00 vs. 41.36, *p* = 0.003. The KIDESCREEN-10 scores were not significantly different between the victimized and non-victimized groups of neglect (35.50 vs. 41.30, *p* = 0.25).

## 4. Discussion

We developed the FPS-J, the first Japanese scale to measure family poly-victimization and tested its validity using external criteria from the perspective of parents. The results of this study confirmed the validity of the IPV modules against respondents and CAN by respondents, except for the items of sexual abuse and neglect in the CAN module of the FPS-J, using external criteria for IPV and CAN and other scales assessing health among respondents and children. However, this study did not report the criterion-related validity of the EA module of the FPS-J using the gold standard EA.

This study developed the FPS-J with adaptations to the culture and features of family violence in Japan through careful discussions among experts on family violence, such as IPV, CAN, and EA, and through cognitive interviews with 11 care providers and survivors of family violence. Changing to easier wording and adding indications of the pronunciation of advanced Chinese characters to the FPS-J may enhance the availability of this scale for a wider population with diverse bio-psycho-social backgrounds, including persons with impaired cognitive/intellectual abilities due to congenital reasons and/or adverse experiences that may place them at a higher risk of family violence and its poly-victimizations [2,35]. In addition, we carefully and repeatedly held discussions on the definitions of violence/abuse regarding what kinds of actions/behaviors are defined as IPV, CAN, and EA from the perspectives of victims of each violence/abuse (i.e., females and males, children, and elderly people) and added more detailed examples of common forms of violence to the FPS-J (see Appendix A). In Japan, the general public, especially victims and perpetrators of family violence, may tend to underestimate family violence due to Japanese cultural norms, such that family violence is a shameful and private issue in the family and there is a lack of knowledge regarding family violence [36,37]. Adding these examples to the FPS-J may contribute to not only increasing the validity and usability of the FPS-J used in Japan but also afford respondents an opportunity to increase their knowledge regarding family violence and note victimization and/or perpetration by answering the FPS-J. Finally, we made a major revision of the FPS-J by adding items regarding financial violence to the two modules of violence against respondent and partner (IPV victimizations and perpetrations) of the original version of the FPS. The difference in social and economic status between males and females is still significant in Japan, and the Global Gender Gap Report in 2021 reported that Japan will rank 120th out of 150 countries in the world in the Gender Gap Index, the lowest ranking among developed countries [38]. The care providers and survivors interviewed in this study suggested that financial violence is a core component of IPV in Japan because Japanese victims of IPV, especially female victims, are more likely to suffer financially through strong financial controls by perpetrators, such as not being given the money needed to live and being strongly prohibited from working. Their financial struggles often trigger them to seek help [39]. Thus, adding items to IPV modules regarding financial violence should help professionals measure the phenomenon of IPV in Japan more accurately, which should contribute to the development of the FPS-J to better adapt to Japanese culture and situations regarding family violence, which should be useful for the Japanese population. This development of the first Japanese scale to measure the three types of violence (i.e., IPV, CAN, and EA) and poly-victimization in families could contribute to identifying more multidimensional and actual situations of family violence, its risk factors, and developing more effective policies, systems, and interventions to prevent and terminate family poly-victimization in Japan.

This study confirmed the validity of the items regarding IPV against respondents and CAN by respondents, except for the items of sexual abuse and neglect in the CAN module, to which no one reported “yes (victimized)”, in comparison with the external criteria for IPV and CAN (i.e., J-CTS2SF and J-CTS-PC). In addition, the results of this study also show that the two modules of violence against respondent and child showed acceptable validity compared with other external criteria, such as the PCL5-J, K6-J, and J-KIDSCREEN-10. These results indicate that the FPS-J is a useful and effective tool for measuring and assessing violence against respondents, including IPV and CAN. It has been recognized that asking only a single item for each form of violence/abuse makes it difficult to evaluate the phenomenon of IPV and CAN [17]; however, showing examples of violence below each single item of the IPV and CAN modules in the FPS-J should encourage respondents to remember their own and their children’s experiences in detail and answer more accurately. Previous scales developed to assess IPV and CAN, such as the J-CTS2SF, J-CTS-PC, and Violence Against Women Screen [40], have approximately 10 to 30 items asking directly about detailed experiences of violence, which may lead to more physical and psychological burdens among Japanese respondents who may be more likely to have psychological barriers to disclosing their own experiences and their family’s victimization of violence [39]. As the FPS-J assesses only four items regarding IPV and CAN, it should be more useful in future research and in clinical and support settings to evaluate IPV, CAN, and poly-victimization. In addition, the FPS-J asks respondents with multiple children and/or living parents to select only one child and parent and report their victimization. Thus, especially if the respondent is an abuser, they may avoid reporting on their abused child or parent, which may lead to an underestimation of the results. Regardless of its limitations, the FPS-J can be used as an assessment tool in healthcare and social work settings in the community and facilities to easily assess poly-victimization among parents with diverse backgrounds and family structures.

This study did not confirm the validity of the items regarding EA in the FPS-J compared to the gold standard for EA (J-MCTS). A possible reason is that the age range of parents that the respondents answered about was relatively young: 70.7% of them were under 75 years old, and these parents may be healthier and more independent in daily life and thus less likely to need care or aid from family members, including the respondents of this study and may not be able to be generalized to an elder population in Japan: the average life expectancy in Japan is 84.3 years old, which is the highest in the world [41]. This may also reflect the criteria of respondents to the FPS-J (i.e., parents with children aged 0–18 years), as the participants in this study may be younger overall (the average age was 41 years), thus perhaps directly leading to a younger age of the parents in this study. The Japanese government has defined an intentional act or failure to act that causes or creates a risk of harm to an older adult aged 65 or older as EA and reported that almost most victims of EA were aged over 75 years old (81.6%), had higher levels of dementia and care required, and a lower degree of independence in daily life was associated with a higher degree of EA [34]. This indicates that the FPS-J and J-MCTS could not sufficiently assess EA, and the validity of the EA module in the FPS-J could not be confirmed in this study. Another reason may be the recent features of the family structure in Japan. A government survey in 2020 [42] found that 82.7% of households with a children aged under 18 years old were nuclear families, meaning households with parents/parent and child/children. Thus, the participants in this study might not have been likely to live with their parents; however, the proportion of participants living with their parents in the FPS-J in this study was not identified. A national survey in Japan reported that 87.0% of victims of EA were living together with their abusers (family caregivers) [6]. These circumstances might have led to a higher rate of “don’t know/don’t want to answer” on the items of the EA modules in the FPS (i.e., 24.1% to 32.7%), which might have affected the results regarding the validity of the EA module in the FPS-J.

### Study Limitations

This study had several limitations. First, the participants in this study may have shown a social desirability bias; thus, this study might have underestimated the actual figures. Because of the recruitment method of this study (i.e., random recruitment using the BRR), some features of the participants tended to be close to the features of general population in Japan, such as average annual income of households with a child (USD 74,500) and rate of mental distress (10.3%), according to the Comprehensive Survey of Living Conditions in 2019 [33]. However, the study participants did not include foreign citizens because of the exclusion criteria (i.e., insufficient ability in Japanese), and citizens who experienced violence, especially those who committed CAN and EA, might not have been willing to cooperate with this study because of the reporting policies described in the explanations. Thus, the results of this study, especially the rates/scores of CAN and EA, may have been underestimated and should be interpreted with caution. In addition, this study showed a low response rate (22.6%), which might be influenced by the ethical considerations (i.e., reporting violence detected from their responses to the public facilities). This indicates that persons with a high risk of family violence who were afraid to be reported may not have participated in this study; thus, the results may have been underreported.

Second, this study did not test the validity of all the modules and items of the FPS-J, and tested only those for IPV against respondents and CAN and EA by respondents because of the limitation of existing scales that might be used as external criteria, and only asked respondents about IPV, CAN, and EA. In addition, because the cut-off points of the external criteria of IPV, CAN, and EA (J-CTS2SF, J-CTS-PC, and J-MCTS) were not identified, the accuracy of the FPS-J as a screening tool, including sensitivity and specificity, could not be examined in this study. A further study to examine the validities of the other modules and items of the FPS-J and accuracy of the FPS-J might be necessary in the future.

Third, the participants might have underreported their experiences of violence and/or witnessing violence using the FPS due to the ways of asking about them, especially psychological and physical violence/abuse among their family members, such as their children, parents, and partners. The items of the FPS-J asking whether their family members have been psychologically/physically hurt have led to underreporting because if respondents did not feel or want to believe that what their family members (e.g., child and parent) had experienced constituted being hurt, they might have tended to answer “No” to these items. In particular, perpetrators of violence tend to underestimate their violence because of feelings of guilt and the justification of violence [43]. In addition, items for violence against respondents (e.g., whether they have been psychologically/physically hurt or sexually assaulted) might also have led to underreporting because victims of violence tend to have feelings of being frozen/paralyzed and deny/minimize their victimization to survive in such situations [43,44]. Thus, the results collected from the FPS-J should be interpreted with caution, and further studies that include external criteria from children and the elderly are necessary to test the validity of the FPS-J from more perspectives.

Fourth, there was a possibility that, if they had multiple children and parents, participants would choose a non-abused child and/or parent for the CAN and EA modules of the FPS-J to avoid being reported, although the FPS-J asked them to choose a child randomly according to their birthday. Therefore, the results for the CAN and EA modules may have been underestimated.

Fifth, approximately one-third (24.1–32.7%) of the participants answered “Don’t want to answer/don’t know” in the module on violence against parents. In addition to cases of elderly abuse by the participants, they might not grasp the history or experiences of violence against their parents because they live in households separate from their parents. In addition, approximately 70% (70.5%) of the participants answered about a parent aged < 75 years old for the items of EA. Because the parents to whom the participants answered tended to be younger, more independent, and with less need for care and support than elderly people in the general population, the status and validity of the module of EA of FPS-J abuse might not have been evaluated accurately in this study.

Despite these limitations, this study is the first to develop a scale to assess family poly-victimization more easily and to confirm the validity of the parts of this study. Future studies should include a more vulnerable population with a high risk of family violence, such as children, the elderly, and adults with lower socioeconomic status, and examine the validity of the other modules and items of the FPS-J. Its accuracy is necessary to increase the validity and usability of this scale.

## 5. Conclusions

We developed the FPS-J, adapted it to Japanese culture and features of family violence, and conducted a cross-sectional study using an online self-report questionnaire for parents with children aged less than 19 years to test its validity. This study confirmed the criterion-related validity of the IPV items against respondents and CAN by FPS-J respondents against external criteria for IPV and CAN (J-CTS2SF and J-CTS-PC, respectively). In addition, the validity of the modules of violence against respondents, including IPV and the CAN, were confirmed in the FPS-J for the scales assessing PTSD, depression/anxiety, and child’s HQOL. This study suggests the suitability of the FPS-J for evaluating family poly-victimization within the family, and the validity of parts of the FPS-J, especially those regarding IPV against respondents and CAN by respondents.

## Figures and Tables

**Table 1:** Descriptions of Demographics (*n* = 483).

Variables	Total	
	Mean/*n* (SD/%)	Min–Max
Age	41.71 (7.18)	20–61
Sex		
Male	231 (47.8)	
Female	252 (52.2)	
Nationality		
Japanese	483 (100.0)	
Marital status		
Married	450 (93.2)	
Non-married	33 (6.8)	
Education		
Junior high school graduate	10 (2.1)	
High school graduate	70 (16.4)	
Technical or junior college graduate	117 (24.2)	
University graduate	231 (47.8)	
Graduate degree	46 (9.5)	
Working status		
Working	384 (79.5)	
Non-working	69 (14.3)	
Temporary suspension	30 (6.2)	
Annual income ^a^		
Less than $10,000	7 (1.4)	
$10,000 to less than $25,000	7 (1.4)	
$25,000 to less than $50,000	65 (13.5)	
$50,000 to less than $100,000	220 (45.5)	
More than $100,000	179 (37.1)	
Missing	5 (1.0)	
History of mental illness		
No	416 (86.1)	
Yes	52 (10.8)	
Don’t want to answer	15 (3.1)	
Continued mental illness to now (*n* = 52)		
No	14 (26.9)	
Yes	37 (71.2)	
Missing	1 (1.9)	
History of physical illness		
No	398 (82.4)	
Yes	80 (16.6)	
Don’t want to answer	5 (1.0)	
Continued physical illness to now (*n* = 80)		
No	50 (62.5)	
Yes	29 (36.5)	
Missing	1 (2.0)	
Number of family members living together	3.95 (0.97)	2–8
Number of children	1.90 (0.76)	1–4
1	156 (32.3)	
2	231 (47.8)	
3	85 (17.6)	
4	11 (2.3)	

^a^ = one dollar is approximately 120 yens.

**Table 2:** Descriptions of the Japanese version of the Family Poly-victimization screen (FPS-J) (*n* = 483).

Variables	Total	
	*n* (%)	Min–Max
Violence against respondent		
Psychological violence		
Yes	234 (48.4)	
No	241 (49.9)	
Don’t want to answer/don’t know	8 (1.7)	
Perpetrators		
My partner (IPV)	114 (23.6)	
Physical violence		
Yes	117 (24.2)	
No	361 (74.7)	
Don’t want to answer/don’t know	5 (1.0)	
Perpetrators		
My partner (IPV)	11 (2.3)	
Sexual violence		
Yes	41 (8.5)	
No	431 (89.2)	
Don’t want to answer/don’t know	11 (2.3)	
Perpetrators		
My partner (IPV)	9 (1.9)	
Financial violence		
Yes	46 (9.5)	
No	432 (89.5)	
Don’t want to answer/don’t know	5 (1.0)	
Perpetrators		
My partner (IPV)	8 (1.7)	
CAN		
Child’s age	9.70 (5.32)	1–19
Psychological abuse		
Yes	169 (35.0)	
No	283 (58.6)	
Don’t want to answer/don’t know	31 (6.4)	
Perpetrators		
Respondent	108 (22.4)	
Physical abuse		
Yes	70 (14.5)	
No	396 (82.0)	
Don’t want to answer/don’t know	17 (3.5)	
Perpetrators		
Respondent	28 (5.6)	
Sexual abuse		
Yes	5 (1.0)	
No	471 (97.5)	
Don’t want to answer/don’t know	7 (1.4)	
Perpetrators		
Respondent	0 (0.0)	
Neglect		
Yes	2 (0.4)	
No	477 (98.9)	
Don’t want to answer/don’t know	4 (0.8)	
Perpetrators		
Respondent	0 (0.0)	
Violence against a parent (Elder abuse)		
A parent is alive: yes	468 (96.9)	
Parent who you answered about (*n* = 468)		
My mother	341 (72.9)	
My father	95 (19.7)	
My mother-in-low (my partner’s mother)	21 (4.3)	
My father-in-low (my partner’s father)	11 (2.3)	
Parent’s age (*n* = 468)		
Less than 65 years old	120 (25.6)	
65 to 74 years old	210 (44.9)	
More than 75 years old	138 (29.5)	
Psychological abuse (*n* = 468)		
Yes	111 (23.7)	
No	228 (48.7)	
Don’t want to answer/don’t know	129 (27.6)	
Perpetrators		
Respondent	11 (2.3)	
Physical abuse (*n* = 468)		
Yes	38 (8.1)	
No	300 (64.1)	
Don’t want to answer/don’t know	130 (27.8)	
Perpetrators		
Respondent	2 (0.6)	
Sexual abuse (*n* = 468)		
Yes	1 (0.2)	
No	314 (67.1)	
Don’t want to answer/don’t know	168 (32.7)	
Perpetrators		
Respondent	0 (0.0)	
Neglect (*n* = 468)		
Yes	4 (0.9)	
No	342 (73.1)	
Don’t want to answer/don’t know	137 (26.1)	
Perpetrators		
Respondent	0 (0.0)	
Financial abuse (*n* = 468)		
Yes	28 (6.0)	
No	327 (69.9)	
Don’t want to answer/don’t know	128 (24.1)	
Perpetrators		
Respondent	0 (0.0)	

Note. IPV = intimate partner violence; CAN = child abuse.

**Table 3:** Descriptions of the variables used as external criterions (*n* = 483).

Variables	Total	
	Mean/*n* (SD/%)	Min–Max
J-CTS2SF (IPV to respondent)		
Psychological aggression	2.19 (6.17)	0–50
Physical assault	0.27 (2.13)	0–40
Injuries	0.08 (0.49)	0–4
Sexual coercion	0.32 (2.80)	0–50
J-CTS-PC (CAN by respondent)		
Psychological aggression	10.16 (15.44)	0–100
Physical assault_total	3.98 (13.44)	0–200
Physical assault_minor	2.98 (9.06)	0–100
Physical assault_severe	0.90 (5.35)	0–100
Physical assault_extreme	0.46 (2.88)	0–50
Sexual abuse	3.74 (8.52)	0–75
Neglect	0.02 (0.24)	0–4
J-MCTS (Elder abuse by respondent)		
Psychological mistreatment	0.29 (1.16)	0–17
Physical mistreatment	0.06 (0.96)	0–20
K6-J	2.52 (3.50)	0–24
Rate		
Psychological distress	91 (18.8)	
Mood/anxiety disorder	34 (7.0)	
Severe mental disorder	13 (2.7)	
PCL5-J	6.69 (9.85)	0–54
Severe PTSD symptoms (>30) ^a^	23 (4.8)	
J-KIDSCREEN-10 (*n* = 255)	41.11 (5.82)	17–50

Note. IPV = intimate partner violence; CAN = child abuse; PTSD = posttraumatic stress disorder; J-CTS2SF = The Japanese version of the Conflict Tactics Scale Short Form; J-CTS-PC = The Japanese version of the Conflict Tactics Scale Parent Child; J-MCTS = The Japanese version of the Modified Conflict Tactics Scale; PCL5-J = The Japanese version of the PCL5; K6-J = The Japanese version of the Kessler 6; J-KIDSCREEN-10 = The Japanese version of the KIDSCREEN-10. ^a^ The cutoff point over 31 was classified as having severe PTSD symptoms.

**Table 4:** Criterion related validity of The Japanese version of the Family Poly-victimization Screen (FPS-J; *n* = 483).

Gold Standards	FPS Items for IPV, CAN, and Elder Abuse		
	Victimized	Non-Victimized	
	Mean (SD)	Mean (SD)	*r*
J-CTS2SF (IPV to respondent)			
Psychological aggression	7.46 (10.23) ^b^	0.56 (2.72) ^b^	<0.001
Physical assault	4.00 (4.95) ^c^	1.77 (1.96) ^c^	<0.001
Injuries	1.27 (1.49) ^d^	0.05 (3.67) ^d^	<0.001
Sexual coercion	8.89 (15.81) ^e^	0.12 (1.35) ^e^	<0.001
J-CTS-PC (CAN by respondent)			
Psychological aggression	21.32 (19.85) ^f^	6.63 (12.22) ^f^	<0.001
Physical assault_total	20.21 (24.32) ^g^	3.11 (12.13) ^g^	<0.001
Physical assault_minor	14.44 (20.28) ^h^	2.36 (7.50) ^h^	<0.001
Physical assault_severe	5.04 (7.21) ^i^	0.66 (6.21) ^i^	<0.001
Physical assault_extreme	2.54 (5.32) ^j^	0.34 (2.55) ^j^	<0.001
Sexual abuse	NA ^a^	NA ^a^	NA ^a^
Neglect	NA ^a^	NA ^a^	NA ^a^
J-MCTS (Elder abuse by respondent)			
Psychological mistreatment	0.55 (1.29) ^k^	0.26 (1.20) ^k^	0.44
Physical mistreatment	NA ^a^	NA ^a^	NA ^a^

Note. IPV = intimate partner violence; CAN = Child abuse; J-CTS2SF = The Japanese version of the Conflict Tactics Scale Short Form; J-CTS-PC = The Japanese version of the Conflict Tactics Scale Parent Child; J-MCTS = The Japanese version of the Modified Conflict Tactics Scale; Victimized = Answered “yes” on an item for each violence/abuse in the FPS-J; Non-victimized = Answered “No” on an item for each violence/abuse in the FPS-J. ^a^ 1 Because none answered “yes”, a score of CTS-PC/MCTS couldn’t be compared between the two groups; ^b^ Psychological violence (FPS_IPV): *n* = 112 (Victimized) vs. *n* = 355 (Non-victimized); ^c^ Physical violence (FPS_IPV): *n* = 11 (Victimized) vs. *n* = 462 (Non-victimized); ^d^ Physical violence (FPS_IPV): *n* = 11 (Victimized) vs. n = 462 (Non-victimized); ^e^ Sexual violence (FPS_IPV): *n* = 9 (Victimized) vs. *n* = 456 (Non-victimized); ^f^ Psychological abuse (FPS_CAN): *n* = 105 (Victimized) vs. *n* = 328 (Non-victimized); ^g^ Physical abuse (FPS_CAN): *n* = 24 (Victimized) vs. *n* = 400 (Non-victimized); ^h^ Physical abuse (FPS_CAN): *n* = 25 (Victimized) vs. *n* = 415 (Non-victimized); ^i^ Physical abuse (FPS_CAN): *n* = 26 (Victimized) vs. *n* = 413 (Non-victimized); ^j^ Physical abuse (FPS_CAN): *n* = 28 (Victimized) vs. *n* = 424 (Non-victimized); ^k^ Psychological abuse (FPS_elder abuse): *n* = 11 (Victimized) vs. *n* = 317 (Non-victimized).

## Data Availability

The data presented in this study are available on request from the corresponding author.

## Appendix A

**Table A1:** Items of the Japanese Version of the Family Polyvictimization Scale (FPS-J).

Module 1	**IPV (respondent’s experience)**
1.	Have you been psychologically hurt?
2.	Have you been physically hurt?
3.	Has anyone forced you to engage in sexual activities?
4.	Have you experienced financial violence?
Module 2	**IPV (partner’s experience)**
5.	Has he/she been psychologically hurt by you?
6.	Has he/she been physically hurt by you?
7.	Has anyone forced him/her to engage in sexual activities?
8.	Has he/she experienced financial violence?
Module 3	**CAN**
9.	Has he/she been psychologically hurt by anyone?
10.	Has he/she been physically hurt by anyone?
11.	Has he/she experienced sexual abuse by anyone?
12.	Has he/she been neglected?
Module 4	**EA**
13.	Has he/she been psychologically hurt by anyone?
14.	Has he/she been physically hurt by anyone?
15.	Has anyone forced him/her to engage in sexual activities?
16.	Has he/she been neglected?
17.	Has he/she ever been robbed of his/her property?

Note. Examples provided for psychological hurt included yelling, shaming, monitoring, isolating from others, threatening to hit, threatening to throw something, destroying belongings, witnessing IPV (CAN), unnecessary restriction of physical freedom (EA) etc. Examples provided for physical hurt included hitting or slapping, shaking, dousing with cold water, throwing something, pushing or shoving, grabbing, dragging hair, beating or kicking, choking, burning or scalding, injuring, etc. Examples provided for forced sexual activities included having sexual intercourse or touching private parts against one’s wishes, sexual harassment, ignoring one’s request to use a condom, taking nude photos against one’s will, inappropriate sexual contact in bathrooms (CAN), forcing the child to bathe together for early adolescent children (CAN) etc. Examples provided for neglect included deprived of care and sufficient food, lacking a safe place to stay, lacking the opportunities to receive necessary health services and education (EA/CAN), etc. Examples provided for financial violence (IPV) included not being given living money, being prohibited from working, etc. Examples provided for financial exploitation (EA) included money taken without one’s permission, being cheated or put under pressure to sign any financial documents, etc.
